# Ultrasound assessment of muscle mass in response to exercise training in chronic kidney disease: a comparison with MRI

**DOI:** 10.1002/jcsm.12429

**Published:** 2019-05-03

**Authors:** Douglas W. Gould, Emma L. Watson, Thomas J. Wilkinson, Joanne Wormleighton, Soteris Xenophontos, Joao L. Viana, Alice C. Smith

**Affiliations:** ^1^ Department of Health Sciences University of Leicester Leicester UK; ^2^ Department of Cardiovascular Sciences University of Leicester Leicester UK; ^3^ Department of Imaging Glenfield Hospital Leicester UK; ^4^ Department of Respiratory Sciences University of Leicester Leicester UK; ^5^ Research Center in Sports Sciences, Health Sciences and Human Development CIDESD, University Institute of Maia ISMAI Maia Portugal

**Keywords:** Muscle wasting, Ultrasound, MRI, Measurement, Chronic kidney disease

## Abstract

**Background:**

Chronic kidney disease (CKD) is a catabolic condition associated with muscle wasting and dysfunction, which associates with morbidity and mortality. There is a need for simple techniques capable of monitoring changes in muscle size with disease progression and in response to interventions aiming to increase muscle mass and function. Ultrasound is one such technique; however, it is unknown how well changes in muscle cross‐sectional area (CSA) measured using ultrasound relate to changes in whole muscle volume measured using magnetic resonance imaging. We tested whether rectus femoris CSA (RF‐CSA) could be used as a valid indication of changes in quadriceps muscle volume as a single measure of muscle size and following a 12 week exercise intervention that resulted in muscle hypertrophy.

**Methods:**

Secondary analysis of data was collected from the ExTra CKD study (ISRCTN 36489137). Quadriceps muscle size was assessed from 36 patients with non‐dialysis CKD before and after 12 weeks of supervised exercise that resulted in muscle hypertrophy.

**Results:**

Strong positive correlations were observed between RF‐CSA and quadriceps volume at baseline (*r* ^2^ = 0.815, CI 0.661 to 0.903; *P* < 0.001) and following 12 week exercise (*r* ^2^ = 0.845, CI 0.700 to 0.923; *P* < 0.001). A moderate positive association was also observed between changes in RF‐CSA and quadriceps following exercise training (rho = 0.441, CI 0.085 to 0.697; *P* = 0.015). Bland–Altman analysis revealed a small bias (bias 0.6% ± 12.5) between the mean percentage changes in RF‐CSA and quadriceps volume but wide limits of agreement from −24 to 25.

**Conclusions:**

Rectus femoris CSA appears to be a reliable index of total quadriceps volume as a simple measure of muscle size, both as a single observation and in response to exercise training in non‐dialysis CKD patients.

## Introduction

Chronic kidney disease (CKD) is a global health problem estimated to affect 8–16% of the population worldwide^1^ and is associated with increased morbidity and mortality.^2^ CKD is a catabolic condition characterized by the progressive wasting of skeletal muscle tissue resulting from underlying complications such as acidosis, systemic inflammation, insulin resistance, and increased levels of myostatin .^3^, ^4^, ^5^ Whilst commonly reported in patients receiving chronic haemodialysis (i.e. those with end stage renal disease), the wasting process appears to start early in the disease process^6^, ^7^, ^8^, ^9^ presenting as early as CKD stage 2 (estimated glomerular filtration rate <80 mL/min/kg). Importantly, recent observational studies have shown that the loss of muscle mass is independently associated with mortality, major cardiovascular events, and physical dysfunction in patients with non‐dialysis dependent CKD.^10^, ^11^ It is therefore apparent that muscle wasting encompassing both structural and functional abnormalities is evident amongst CKD populations and associates with poor outcomes.

Consequently, it is important to be able to characterize and quantify these changes in individuals with CKD in order for early recognition of skeletal muscle dysfunction and allowing for the application of appropriate interventions aimed at improving muscle structure and function. Current guidelines for the screening of muscle wasting focus on the use of advance imaging techniques such as dual‐energy X‐ray absorptiometry, magnetic resonance imaging (MRI), and computed tomography. The application of these methods in kidney disease populations has recently been extensively reviewed .^12^

Magnetic resonance imaging is often considered the gold standard for the assessment of muscle size due to its high contrast images that allow for separation of fat, muscle, and bone .^13^ MRI measures of quadriceps cross‐sectional area (CSA) are associated with physical performance methods in dialysis 14 and, more recently, non‐dialysis CKD .^9^ Unfortunately, the use of MRI is limited predominantly to research settings due to the high cost, need for technical expertise required, and time‐consuming image analysis.

Ultrasound imaging is a commonly available technique used within clinical settings; however, its application for the assessment of muscle mass in CKD populations has been largely overlooked in comparison with the aforementioned methods. Despite this, ultrasound imaging allows for a quick and accurate assessment of individual muscle architectural characteristics and has been shown to correlate well with MRI in healthy populations.^13^, ^15^, ^16^ In addition to this, there are now a growing number of studies showing that the architecture of the lower extremities [most commonly the rectus femoris (RF)] assessed by ultrasound is associated with physical performance in older and other clinical populations .^17^, ^18^, ^19^

In CKD populations, ultrasound has recently been used to identify the presence of low muscle mass, measured as quadriceps thickness, in end stage renal disease patients receiving haemodialysis.^20^ We have shown that ultrasound‐derived RF‐CSA is associated with physical performance amongst patients with CKD stages 3b–5 not requiring dialysis.^21^ The utility of ultrasound to detect changes in muscle mass in response to an intervention in CKD populations, in comparison with a gold standard (i.e. MRI), is unknown. Therefore, we aimed to (i) investigate the association between a RF‐CSA assessed using ultrasound and total quadriceps volume measured by the gold standard MRI before and (ii) investigate comparisons between ultrasound and MRI in response to a 12 week exercise intervention that resulted in increased muscle size and volume.^22^

## Material and methods

### Participants

This was a secondary analysis of data collected during the ExTra CKD (ISRCTN: 36489137) conducted at the University Hospitals of Leicester (UHL) NHS Trust between December 2013 and October 2016. This trial investigated the effects of 12 weeks combined aerobic and resistance training compared with aerobic exercise only and reported muscle hypertrophy following both interventions.^22^ Patients gave written informed consent, and the study received ethical approval from the National Research Ethics Committee, East Midlands‐Leicester (Ref: 13/EM/0344), and University Hospitals Leicester (Ref: UHL 137056).

To be eligible for the ExTra CKD trial, participants (i) were diagnosed with moderately severe CKD (stages 3b–5), (ii) were aged ≥18 years, (iii) had no physical impairment and significant co‐morbidities that were a contraindication to exercise [unstable hypertension, potentially lethal arrhythmia, myocardial infarction within previous 6 months, unstable angina, active liver disease, uncontrolled diabetes mellitus (HbA1c >9%), advanced cerebral or peripheral vascular disease), and (iv) had sufficient command of English to give informed consent.

### Quadriceps muscle volume

**Figure 1:**
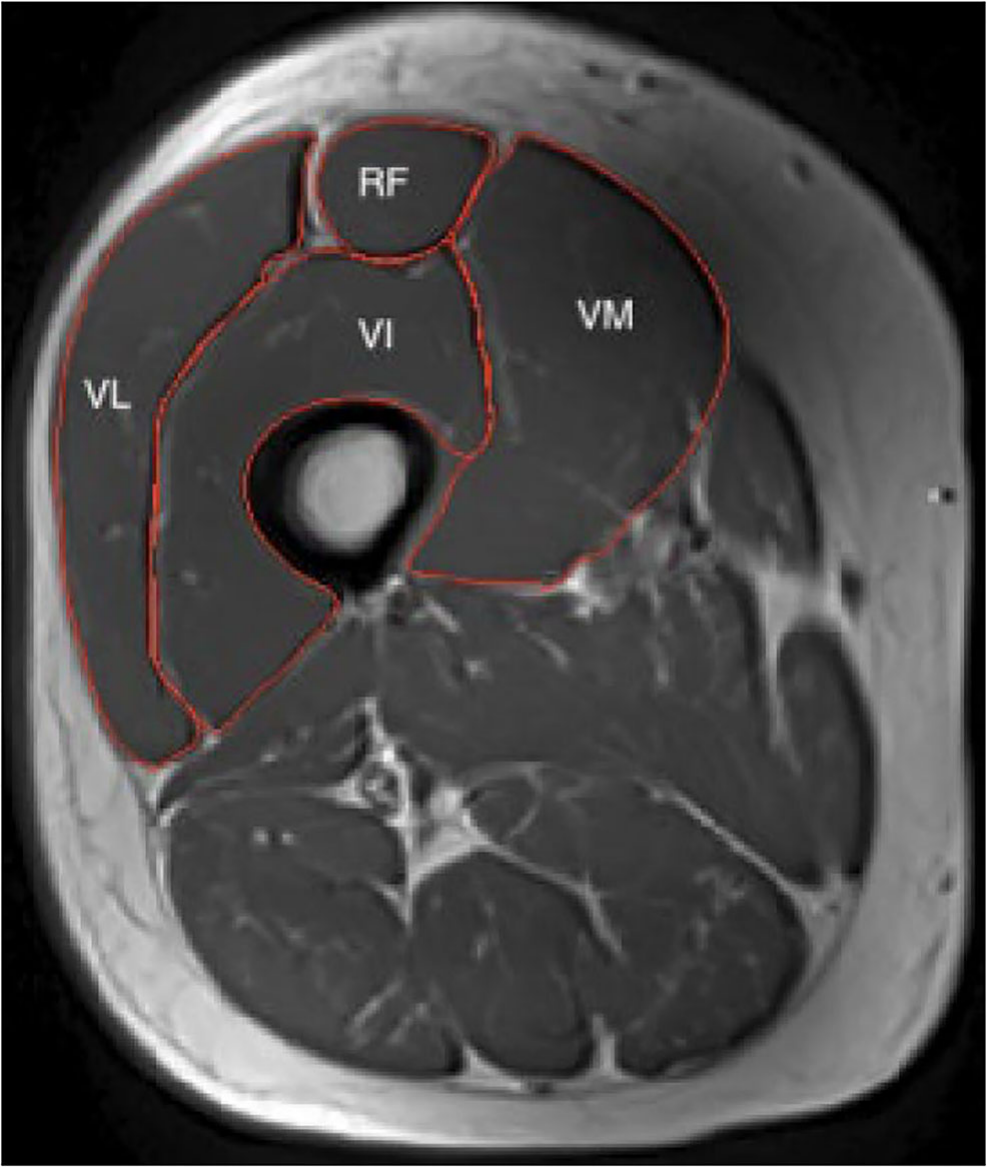
Representative magnetic resonance image of the quadriceps muscle group. Rectus femoris (RF), vastus lateralis (VL), vastus intermedius (VI), and vastus medialis (VM) highlighted.

Images of the quadriceps muscles were acquired from participants' right leg before (baseline) and post the 12 week exercise intervention using a 3 Tesla MRI scanner (Siemens Skyra). Images of the entire thigh (from the proximal border of the patella to the superior aspect of the femur) were obtained in the axial plane using a T1 turbo spin‐echo sequence with the following parameters: slice thickness = 5 mm with no gap between slices; repetition time/echo time = 873 ms/14 ms; field of view = 450 × 309.4 mm; in‐plane resolution = 0.879 × 0.879 mm. Quadriceps volume was measured on 10 mm thick slices by manually outlining the fascial boundary using online imaging analysis software (Xinapse Systems, UK) (*Figure* 1). Where possible, individual muscles were outlined and visible fat or connective tissue within the measurement region avoided. All scans were analysed by a single‐blinded researcher (DWG).

### Rectus femoris anatomical cross‐sectional area

**Figure 2:**
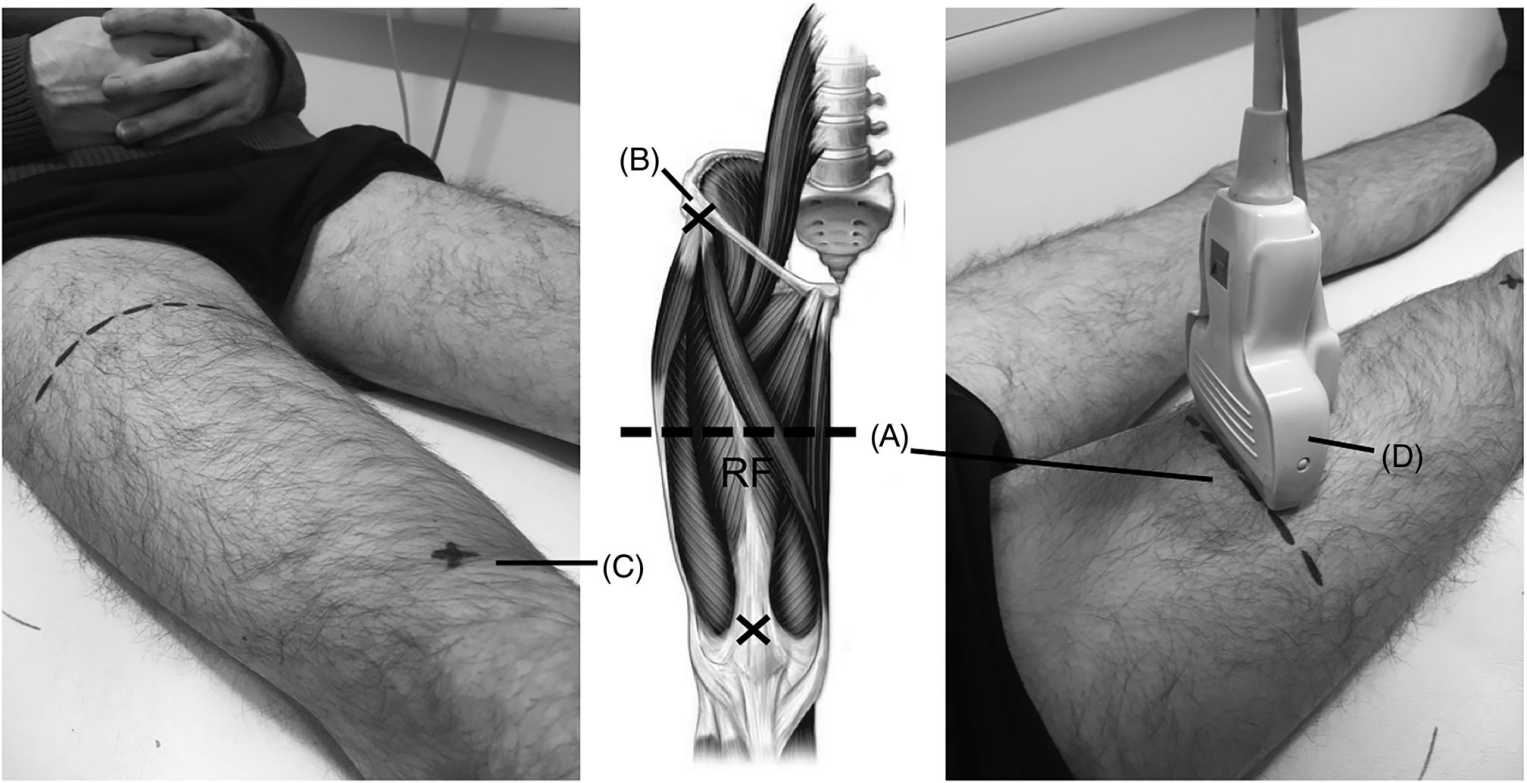
Imaging site and probe placement. Site of scan (A), greater trochanter (B), superior aspect of the patella (C), probe placement (D).

**Figure 3:**
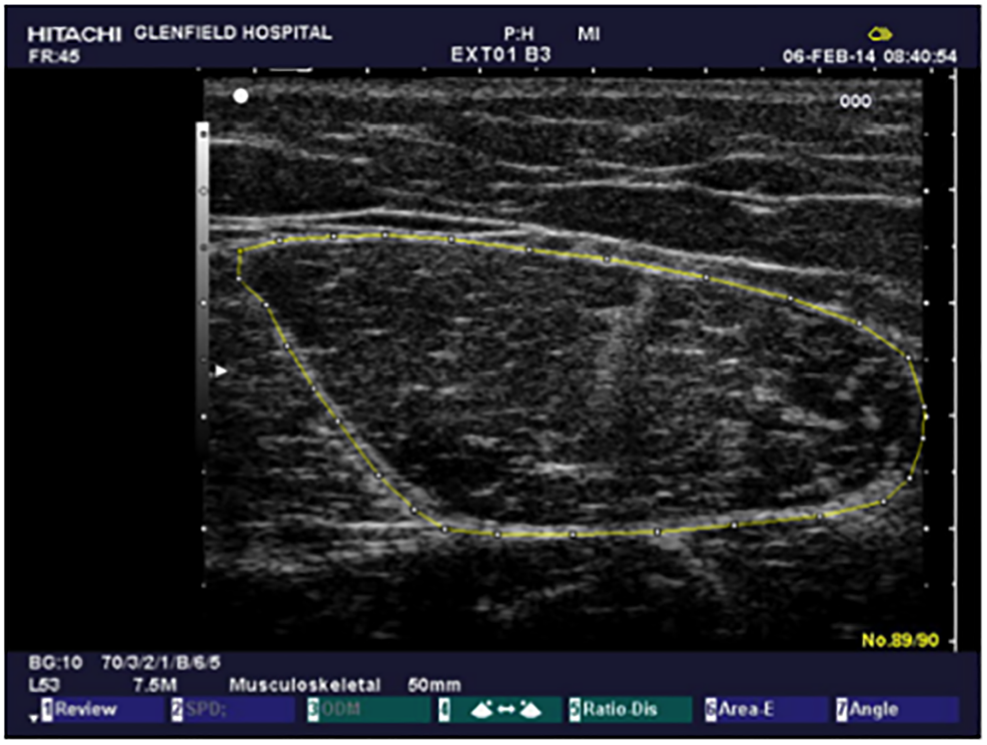
Representative image of rectus femoris cross‐sectional area measured by 2D B‐mode ultrasound.

Rectus femoris anatomical CSA was measured from the right leg using B‐mode 2D ultrasonography (Hitachi EUB‐6500; probe frequency, 7.5 MHz) under resting conditions with the participant lying prone at a 45° angle. Imaging was performed at the midpoint between the greater trochanter and the superior aspect of the patella on the mid‐sagittal plane of the thigh, identified using a measuring tape and marked with anatomical pen. The location of the imaging site of the scan (i.e. distance from the patella on the anterior part of the thigh) was recorded to standardize the measurement site for future scans. To obtain the cross‐sectional image, the probe was placed transversally to the longitudinal axis of the thigh forming a 90° angle to the skin surface (*Figure* 2). Ample contact gel was applied to the area and ultrasound transducer allowing minimal pressure to be applied to the probe to avoid compression of the muscle. Participants were asked to gently contract and relax their quadriceps to delineate parameters of the RF prior to image acquisition. RF‐CSA was then calculated by outlining echogenic fascial line of the RF using the track ball cursor on a frozen image (*Figure* 3). RF‐CSA was calculated as an average of three consecutive measurements with <10% variation. A single researcher (DWG) performed all ultrasound scans with an interclass correlation coefficient determined of 0.95 and was blinded to the baseline values.

### Statistical analysis

The distribution of variables was assessed using the Shapiro–Wilk's test. Associations between MRI‐derived and ultrasound‐derived were assessed using Pearson product‐moment (*r*) or Spearman's rank correlation (*rho*) coefficients accordingly and displayed as scatter plots with 95% confidence intervals (CI). The test–retest reliability of ultrasound measures of RF‐CSA was assessed using the intraclass correlation coefficient with two‐way absolute agreement (represented at *r*). Bland–Altman analysis was performed to assess the agreement between the changes in RF‐CSA and quadriceps volume, when expressed as a percentage. All statistical analysis was carried out using IBM spss Statistics (IBM, Chicago, IL) Version 24 and Prism Version 7 (GraphPad Software, Inc.).

## Results

### Patient characteristics

Out of the 54 patients consented into the main trial, 41 were randomized to receive combined aerobic and resistance exercise or aerobic exercise only. Of these, 36 patients completed the 12 week intervention; of which, 35 underwent both quadriceps MRI and ultrasound scan at baseline and were therefore included in these analyses. Participant baseline characteristics are shown in Table 1. We were unable to obtain post‐exercise MRI data for four participants; therefore, associations between post‐exercise ultrasound‐derived and MRI‐derived muscle size were investigated in 31 participants.

**Table 1:** Participant characteristics

	n = 36
Age (years)	61.6 (±11.8)
Gender, female, *n* (%)	22 (61)
eGFR (mL/min/1.73 m^2^)	25.5 (±7.8)
RF‐CSA (cm^2^)	8.5 (±2.8)
Quadriceps volume (cm^2^)	935.5 (±315.5)
Ethnicity	
White British, *n* (%)	23 (64)
South Asian, *n* (%)	12 (33)
Black Caribbean, *n* (%)	1 (3)
Primary cause of disease	
Diabetic nephropathy, *n* (%)	3 (8)
Interstitial nephritis, *n* (%)	4 (11)
IgA nephropathy, *n* (%)	3 (8)
Polycystic kidney disease, *n* (%)	3 (8)
Other, *n* (%)	2 (6)
Unknown/aetiology uncertain, *n* (%)	21 (58)
Comorbid conditions	
Diabetes, *n* (%)	9 (25)
CVD, *n* (%)	6 (17)

eGFR, estimated glomerular filtration rate; RF‐CSA, rectus femoris cross‐sectional area; IgA, immunoglobulin A; CVD, cardiovascular disease.

### Morphological adaptations to 12 week supervised exercise

As previously reported,^22^ muscle hypertrophy was observed following both combined and aerobic only exercise interventions. When looking at the groups as a whole, 12 week supervised exercise resulted in mean increases of 0.57 cm^2^ (6.7%) (CI 0.27 to 0.86 cm^2^, *P* < 0.001) for RF‐CSA and 65.03 cm^3^ (7.0%) (CI 37.49 to 95.58 cm^3^, *P* < 0.001) for total quadriceps volume.

### Association between rectus femoris cross‐sectional area and total quadriceps volume

**Figure 4:**
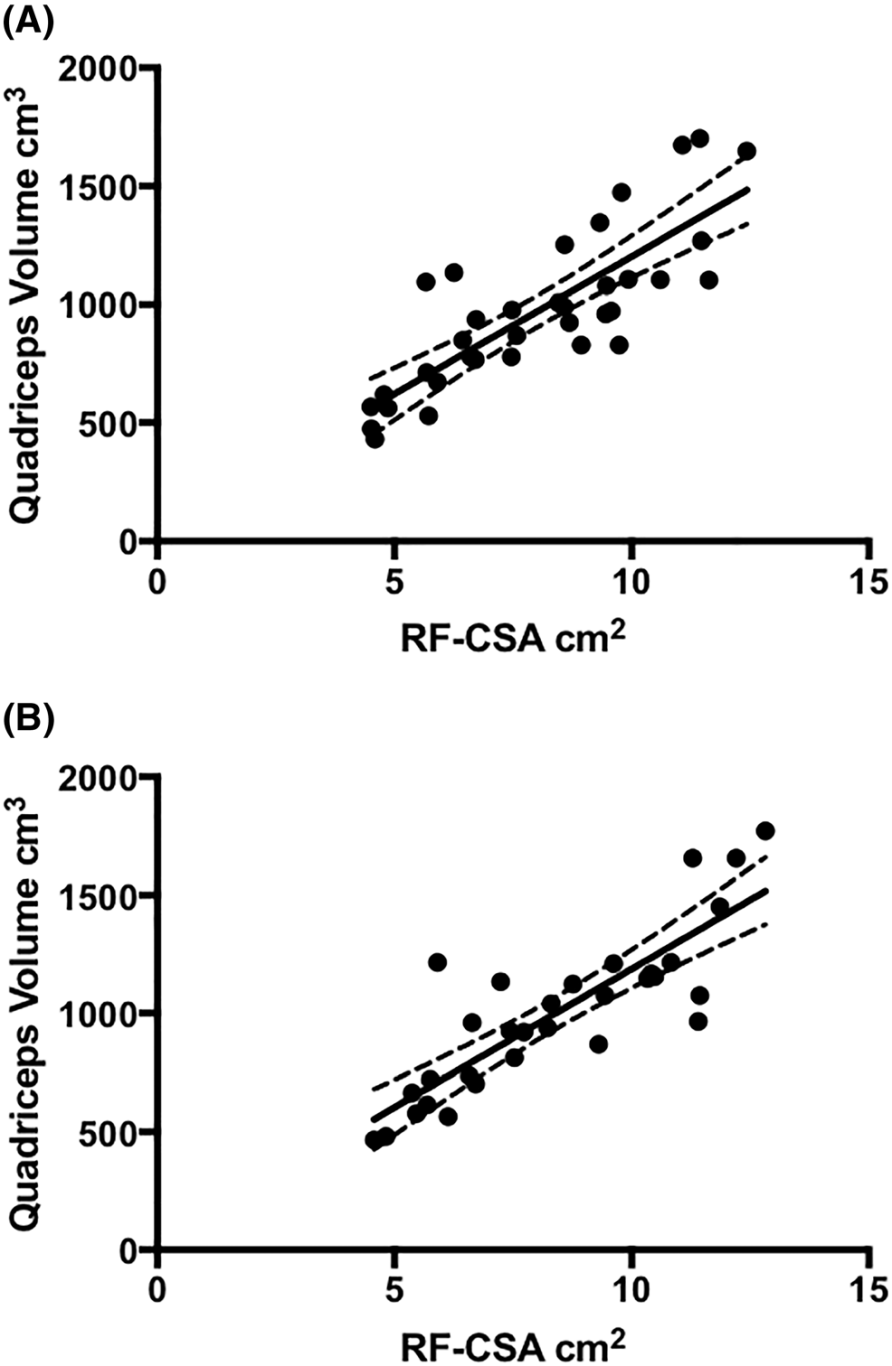
Association between RF‐CSA and quadriceps muscle volume measured at baseline (A) and following a 12 week exercise intervention (B). RF‐CSA, rectus femoris cross‐sectional area.

*Figure* 4 shows the association between MRI‐derived quadriceps volume and ultrasound‐derived RF‐CSA. We observed strong positive correlations between the two measures at baseline (*r* ^2^ = 0.815, CI 0.661 to 0.903; *P* < 0.001) and at the end of the 12 week exercise intervention (*r* ^2^ = 0.845, CI 0.700 to 0.923; *P* < 0.001).

**Figure 5:**
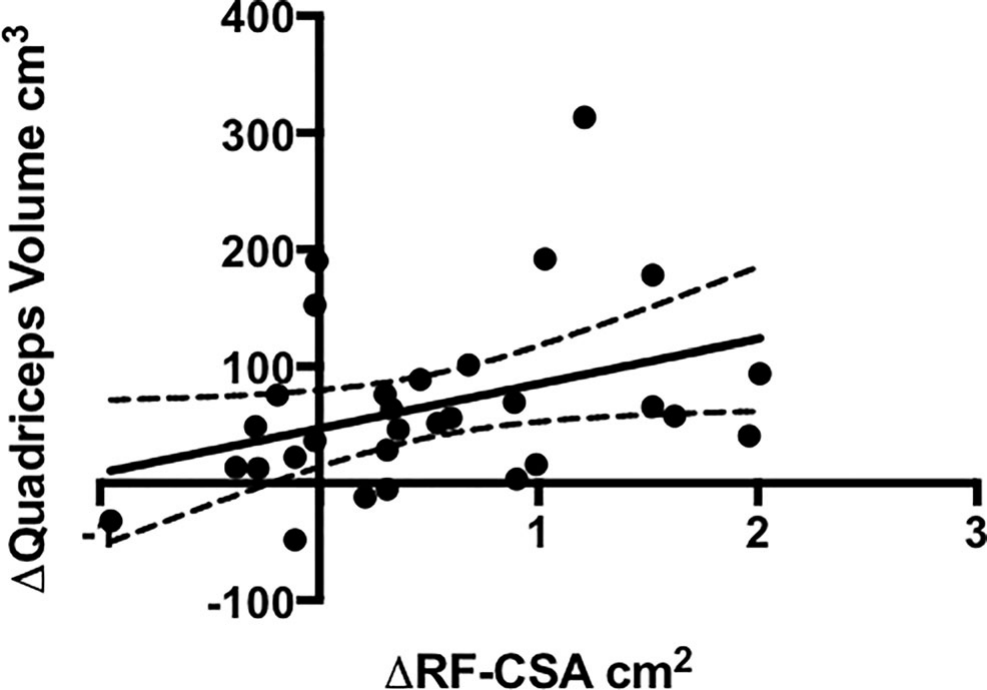
Association between the delta change in RF‐CSA and quadriceps muscle volume following 12 weeks exercise. RF‐CSA, rectus femoris cross‐sectional area.

A moderate positive correlation (rho = 0.441, CI 0.085 to 0.697; *P* = 0.015) was also observed when investigating the associations between delta‐change values (i.e. change in MRI‐derived muscle volume and ultrasound‐derived RF‐CSA), calculated as post‐exercise minus baseline (*Figure* 5).

**Figure 6:**
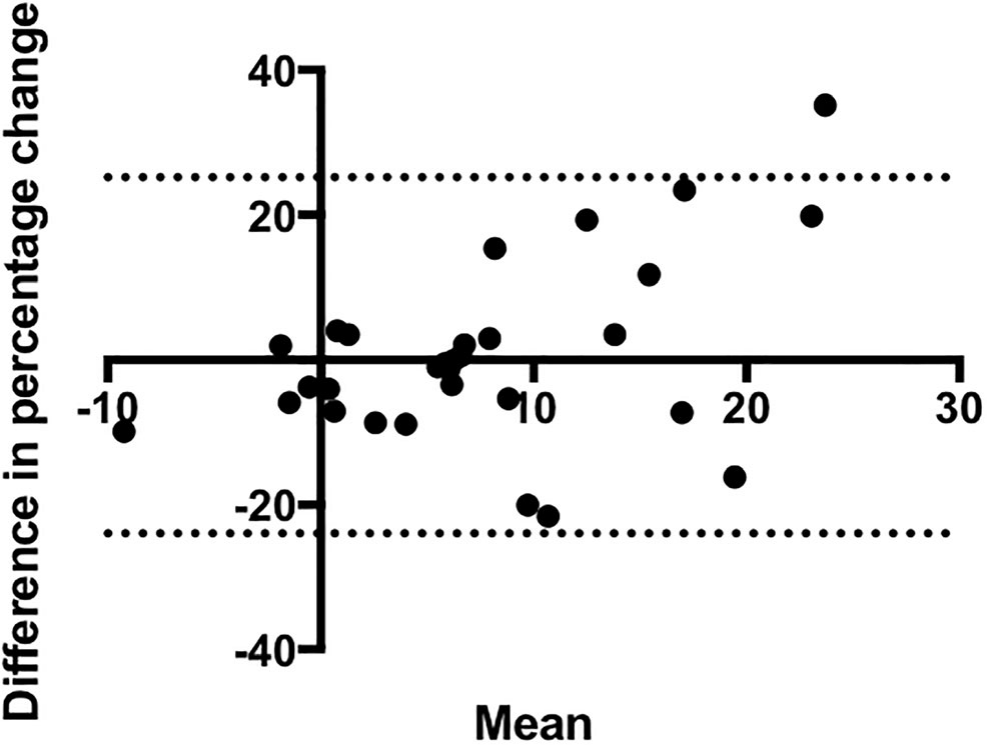
Bland–Altman plot with 95% confidence limits.

When expressing delta‐change values as a percentage, the Bland–Altman analysis (*Figure* 6) showed a small bias between the mean percentage changes in RF‐CSA and quadriceps volume (bias 0.6% ± 12.5) but wide limits of agreement from −24 to 25.

## Discussion

We have demonstrated that ultrasound‐derived RF‐CSA correlates with total quadriceps volume prior to and following a 12 week exercise intervention that resulted in muscle hypertrophy.^22^ Importantly, although to a lesser extent, the change in RF‐CSA over the 12 week exercise intervention also correlated with the change in quadriceps volume. Bland–Altman analysis revealed a small bias (0.6%) between the percentage change in RF‐CSA and quadriceps volume measured by ultrasound and MRI, respectively. However, despite the small difference between percentage changes in RF‐CSA and quadriceps volume, the 95% limits of agreement of −24% to 25% suggest that this varies widely. Taken together, these data suggest that ultrasound‐derived RF‐CSA is sensitive to changes in quadriceps muscle mass over time and in response to an intervention in patients with non‐dialysis CKD.

Low muscle mass has been shown to be independently associated with worse clinical outcomes in CKD populations and is likely a major contributing factor to the reduced physical performance that is commonly reported amongst this group. Importantly, quadriceps muscle size and function is well recognized as an important contributor to reduce exercise capacity and increase morbidity and mortality in chronic disease populations.^9^, ^23^ Indeed, we have recently shown that RF‐CSA measured by ultrasound is independently associated with physical performance measures amongst non‐dialysis CKD patients.^21^ Therefore, being able to quickly and accurately quantify changes in lower limb muscle that are functionally important—such as RF—is of particular interest to clinicians and researchers.

The measurement of muscle size with other imaging techniques (such as dual‐energy X‐ray absorptiometry, computed tomography, and MRI) is time‐consuming, expensive, and requires specialist equipment and operators meaning that they are often not used for clinical evaluation. In contrast, ultrasound offers a quick and relatively simple assessment of muscle size that can be conducted in the majority of clinical settings. Indeed, the evidence supporting the use of ultrasound as a quick and simple tool for the assessment of muscle mass is growing. In agreement with the results presented here, a recent systematic review concluded that ultrasound is a valid and reliable tool for muscle quantification in older adults and some clinical populations .^24^ Moreover, a number of studies have reported good associations between ultrasound‐derived muscle CSA and thickness with volume derived by MRI .^13^, ^16^, ^25^

More recently, Franchi *et al*.^25^ demonstrated that ultrasound‐derived vastus lateralis (VL) muscle thickness was associated with VL anatomical CSA and volume measured using MRI at a single time point. The authors also report a strong positive correlation between changes in VL muscle thickness and CSA but not volume following resistance exercise in young healthy participants. We have previously shown that ultrasound is sensitive to detect change following an 8 week resistance exercise intervention^26^; however, to the best of our knowledge, this is the first study to demonstrate associations between ultrasound and the gold standard MRI in the measurement of changes in muscle size in response to an intervention in a CKD population.

Although ultrasound is an attractive method for measuring muscle size, it is important to consider a number of limitations. Methodological issues such as site of scan,^27^ identification of landmarks,^28^ patient position,^29^, ^30^ and probe placement including angle and force applied^31^ can all impact the accuracy of the results and may contribute to the heterogeneity in the parameters measured and reported in the skeletal muscle ultrasound literature. As such, although ultrasound is a relatively straightforward technique, appropriate training with validation and reliability work should be performed to ensure consistency with measurements.^28^

### Limitations

It is important to consider the data presented here in view of its limitations. As a secondary analysis of published data, no formal power calculation was performed. Whilst having a single‐blinded assessor perform all ultrasound scans and MRI analysis, this negated any intra‐observer differences in interpretation, which was important for quantifying changes in muscle size over the course of the intervention reported elsewhere.^22^ This may have reduced the generalizability of the results presented here, as in clinical practice, it is unlikely that one individual would perform all imaging and analysis. Finally, whilst the quadriceps muscle group are functionally important, associations with clinical outcomes are currently unknown. Future work should seek to determine the prognostic ability of ultrasound measures to predict important clinical outcomes.

## Conclusions

Ultrasound‐derived RF‐CSA appears to be a reliable index of total quadriceps volume as a simple measure of muscle size in patients with non‐dialysis CKD both at baseline and in response to exercise training in non‐dialysis CKD patients. Ultrasound may therefore be a useful clinical tool in quantifying changes muscle size in muscle size in response to targeted interventions.

## Conflict of interest

The authors declare no conflicts of interest.
